# Thyroid scintigraphy of healthy cats using small-field-of-view gamma cameras

**DOI:** 10.3389/fvets.2024.1453441

**Published:** 2024-10-21

**Authors:** Sijin Cha, Yeon Chae, Taesik Yun, Hakhyun Kim, Byeong-Teck Kang

**Affiliations:** Laboratory of Veterinary Internal Medicine, College of Veterinary Medicine, Chungbuk National University, Cheongju, Republic of Korea

**Keywords:** occupational radiation exposure, technetium-99m pertechnetate, radiation exposure, small field of view (SFOV) gamma camera, thyroid scintigraphy

## Abstract

**Introduction:**

Small-field-of-view (SFOV) gamma cameras can offer higher sensitivities than conventional gamma cameras. However, there are currently no reports on the efficacy and safety of thyroid scintigraphy using SFOV gamma cameras in veterinary medicine. Therefore, we aimed to evaluate the efficacy and radiation safety of an SFOV gamma camera for feline thyroid scintigraphy.

**Materials and methods:**

Three veterinary staff members (operator, staff 1, and staff 2) performed thyroid scintigraphy on 10 healthy cats in this study. The operator administered either 2 or 4 mCi of technetium-99m pertechnetate (^99m^TcO^−^_4_) through the cephalic vein. At 20, 40, and 60 min after injection, thyroid images were obtained using a SFOV gamma camera under various acquisition conditions (100,000, 150,000, and 200,000 counts and 30 and 60 s). Thyroid scintigraphy images were analyzed by calculating the thyroid-to-salivary ratios (TSR) and thyroid-to-background ratios (TBR). Surface and ambient radiation were measured hourly from immediately after injection to 6 h. The cumulative occupational radiation doses were measured during the procedure.

**Results:**

The TSR and TBR median values aligned with the previously reported normal range obtained using a large-field-of-view gamma camera. There were no notable differences in TSR and TBR between the two doses of ^99m^TcO^−^_4_, nor across acquisition conditions and timelines. The 4-mCi group consistently emitted more ambient (*p* < 0.05) and surface (*p* < 0.05) radiation than did the 2-mCi group. Staff 1 consistently received higher cumulative radiation doses than did staff 2 and the operator (*p* < 0.05).

**Conclusion:**

The SFOV gamma camera demonstrated adequate image quality for thyroid scintigraphy in healthy cats even with relatively low doses and short acquisition conditions. Radiation exposure during the procedure posed minimal safety concerns. Therefore, the SFOV gamma camera could be a valuable tool for evaluating thyroid glands in cats.

## 1 Introduction

Thyroid scintigraphy is a valuable tool that offers essential insights into both the structure and function of the thyroid gland in feline patients (1, 2). It plays a crucial role in the diagnosis, staging, and management of thyroid disorders in cats.

In previous studies, thyroid scintigraphy was performed in cats by injecting 2–6 mCi of technetium-99m pertechnetate (^99m^TcO^−^_4_) either intravenously or subcutaneously (3–7). Generally, 20 min to 4 h after ^99m^TcO^−^_4_ injection, scintigraphy is performed using a large-field-of-view (LFOV) gamma camera (3–7). Static images are obtained for 100,000–250,000 counts or 60 s (3–7). Conventional LFOV gamma cameras are large and require dedicated scanning rooms. Owing to the bulkiness of the equipment, achieving close contact between the detector and the organ becomes challenging, leading to an increased detector-to-organ distance and reduced sensitivity (8).

The same issue has been raised in human scintimammography. As an alternative, a high-resolution, small-field-of-view (SFOV) gamma camera optimized for breast imaging has been developed (8, 9). The development of the SFOV gamma camera has improved sensitivity for the detection of nonpalpable and subcentimeter breast lesions in several ways (10–12). Such a camera can minimize the distance between the breast and detector to improve resolution. The flexibility in positioning also helps mitigate image interference from neighboring organs like the heart and liver by restricting the field of view exclusively to the lesion (10–12). Therefore, it can be used to examine small organs, such as the thyroid, parathyroid, and gall bladder.

The application of the SFOV gamma camera in human thyroid scintigraphy yields more than a two-fold increase in counts when the same quantity of radiotracer is administered compared with the conventional LFOV gamma camera (11). Consequently, achieving equivalent results with a reduced radiopharmaceutical dosage reduces patient radiation exposure.

Numerous investigations have addressed the issue of occupational radiation exposure of human medical staff to radiopharmaceuticals used for thyroid scintigraphy (13–16). When performing thyroid scintigraphy in veterinary hospitals, veterinary patients pose unique challenges that are distinct from those that come with human patients. Notably, veterinary patients require restraint to maintain their immobility for optimal imaging during acquisition. Naturally, veterinary staff are at higher risk because of their close contact with radiation resources and extended exposure durations. Despite the necessity for safety evaluations of veterinary thyroid scintigraphy, relevant research is currently limited (17, 18).

We hypothesized that the SFOV gamma camera addresses the limitations of the LFOV gamma camera in feline thyroid scintigraphy. Therefore, the primary objective of this study was to evaluate the diagnostic value of feline thyroid scintigraphy conducted using an SFOV gamma camera, specifically focusing on the relative reduction in ^99m^TcO^−^_4_ dosage and shorter acquisition duration. Furthermore, there are currently no reports on the efficacy and safety of thyroid scintigraphy using SFOV gamma cameras in veterinary medicine. Thus, the secondary objective of this study was to assess the radiation safety of feline thyroid scintigraphy using an SFOV gamma camera.

## 2 Materials and methods

### 2.1 Animals

Six female and four male client-owned cats were included in this study (Supplementary Table 1). The cats were determined to be healthy and evaluated as euthyroid based on their medical history, physical examination, complete blood count, serum chemistry, radiography, and serum thyroxine (T4) concentration. The cats were divided randomly into five cats for the 2-mCi group and five cats for the 4-mCi group by ^99m^TcO^−^_4_ injection dose. Consent was obtained in all cases, and the study was approved by the Institutional Animal Care and Use Committee of Chungbuk National University (Cheongju, South Korea; approval number CBNUA-2006-22-01).

### 2.2 Thyroid scintigraphy

**Figure 1:**
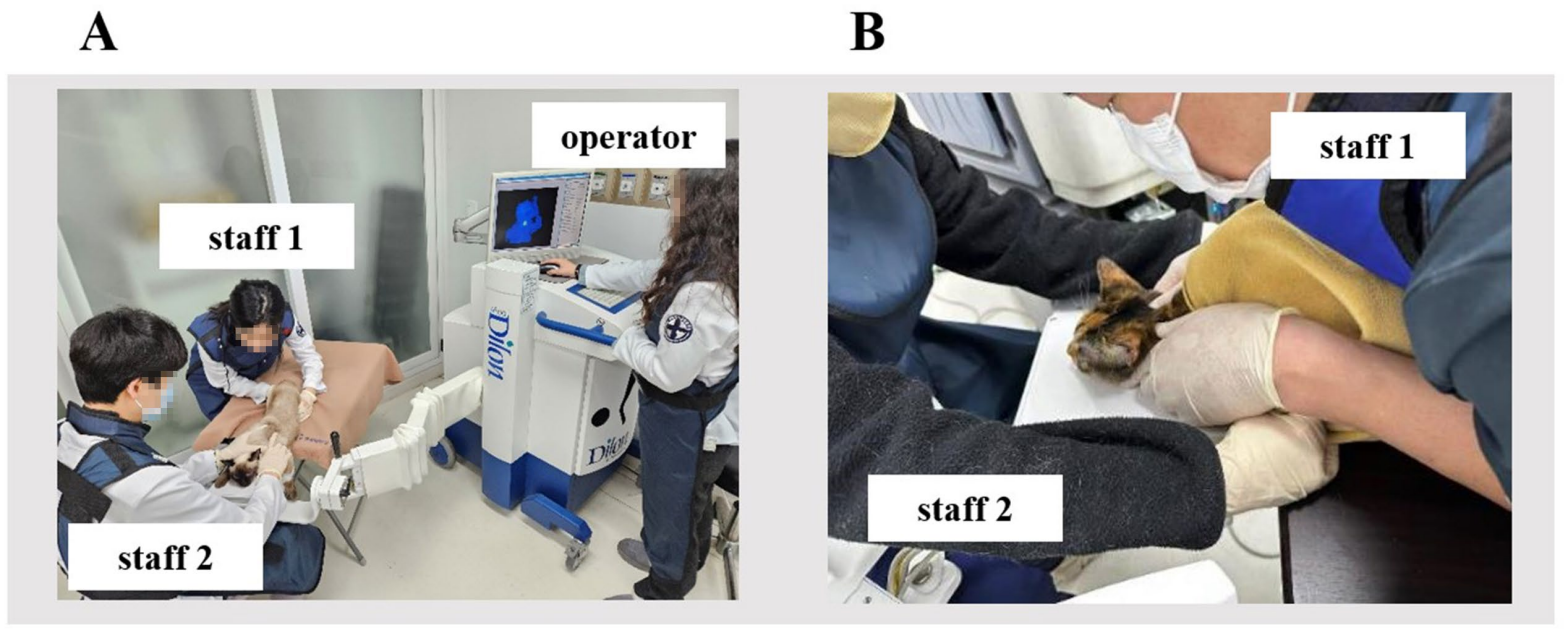
Thyroid scintigraphy in cats using small-field-of-view gamma camera. **(A)** The operator manipulated the gamma camera 150 cm away from the cat. **(B)** Staff 1 held the cat within 20 cm, and staff 2 held the cat’s front legs within 50 cm distance from the waist where the electronic personal dosimeter was worn.

To perform thyroid scintigraphy, three veterinary staff participated in this study: The first (operator) was a veterinarian who manipulated the gamma camera 150 cm away from the cats (Figure 1A). The second (staff 1) was a primary restrainer who held the cats within 20 cm of the waist, where an electronic personal dosimeter (EPD; SPD-9300, Sans Frontier Technology, Seoul, South Korea) was worn (Figure 1). The third (staff 2) served as a secondary restrainer, assisting staff 1 in holding the cats’ front legs within 50 cm of their waist (Figure 1). After staff 1 held the cat properly, the operator injected either 2 mCi or 4 mCi of ^99m^TcO^−^_4_ through the cephalic vein. At 20, 40, and 60 min after injection, the distance between the gamma camera and the source was maintained at less than 100 mm, the cats were placed directly on top of the low-energy all-purpose parallel hole collimator and held in ventral recumbency by staff 1 and 2 (Figure 1B). Thyroid images were obtained with an SFOV gamma camera (Dilon 6800, Dilon Technologies, VA, USA), equipped with a high-resolution parallel-hole collimator and a sodium iodide scintillation detector, which provides spatial resolution of 3.3 mm and an energy resolution of 13.5% (19, 20). The images were acquired by the operator (Figure 1A) and integrated into a dedicated imaging computer running nuclear medicine software (Dilon 6800 software; Dilon Technologies, VA, USA). Static images were obtained under various acquisition conditions in the following order: 100,000 counts (acquisition duration: 17–43 s); 150,000 counts (acquisition duration: 25–64 s); 200,000 counts (acquisition duration: 34–85 s); 30 s; and 60 s.

### 2.3 Image analysis

Thyroid scintigraphy images were analyzed with dedicated nuclear medicine software (Dilon 6800 software; Dilon Technologies, VA, USA) to quantify the level of ^99m^TcO^−^_4_ activity in three regions of interests (ROI): (1) the thyroid lobes, (2) the zygomatic/molar salivary gland, and (3) a background area. The ROI for both the thyroid and salivary glands were meticulously delineated to encompass the entire circumference of each thyroid lobe and the zygomatic/molar salivary gland (4, 21). For background measurements, the axillary region was selected with an ROI approximately equal in size to each thyroid lobe as previously described (22). Ventral thyroid images were employed for this analysis, as they offered superior visualization of the thyroid lobe and salivary gland uptake. To mitigate the potential variability in count density values due to variations in region size, a single operator drew all three ROI.

The mean thyroid-to-salivary ratio (TSR) was determined by dividing the average count density of both thyroid lobes (total thyroid counts/total thyroid pixels) by the mean count density of the salivary glands (total salivary counts/total salivary gland pixels) (3). Similarly, the mean thyroid-to-background ratio (TBR) was determined by dividing the average thyroid count density (total thyroid counts/total thyroid pixels) by the mean background count density (total background counts/total background pixels) (22).

### 2.4 Measurement of radiation exposure

Gamma radiation emission rates were measured at the skin surface (surface radiation; count per second, CPS) and at a distance of 1 m in the horizontal plane from the body (ambient radiation, μSv/h) using a calibrated Geiger–Mueller instrument (Rad 100, International Medcom Inc., CA, USA), which was positioned consistently at the same location to ensure uniform data collection. Radiation emission levels from the cats were checked hourly from immediately after injection up to 6 h.

Each veterinary staff member was assigned EPD to be worn outside of the lead apron on the waist to measure the cumulative occupational radiation dose (μSv). It was selected because of its convenience, sensitivity, and ability to display dose information instantaneously. Specifically, it has a range of 0.1 μSv to 10 Sv and is accurate to within ±10% when properly calibrated.

### 2.5 Statistical analyses

Statistical analyses were performed using IBM SPSS Statistics for Windows (version 25.0; SPSS Inc., Chicago, IL, USA). A *p*-value <0.05 was considered significant. The normality of the distribution of the data was assessed with the Kolmogorov–Smirnov test. Medians (interquartile ranges) were used to represent non-normally distributed data, while means ± standard deviations were used to express normally distributed data. Comparisons of data between the 2-mCi and 4-mCi groups were conducted using the Mann–Whitney U-test. The Kruskal–Wallis test followed by Bonferroni correction of the Mann–Whitney U-test was used to compare radiation emission rates among the three veterinary staff and quantitative scintigraphic parameters among various acquisition conditions (100,000 counts, 150,000 counts, 200,000 counts, 30 s, and 60 s) and different timelines (20 min, 40 min, and 60 min after injection). The changes in ambient and surface radiation over time were analyzed using the Friedman test with Dunn’s multiple comparisons within the same administered activity group. The Friedman test was performed to evaluate differences in the cumulative occupational radiation dose over time within the same staff groups.

## 3 Results

### 3.1 Study group

Ten cats were included in the study. The 2-mCi group consisted of four domestic shorthairs and one Persian shorthair. The 4-mCi group consisted of five domestic shorthairs. There were no significant differences between the two groups concerning age, body weight, sex, or T4 levels (Table 1).

**Table 1:** Demographics of cats included in this study.

	2-mCi group	4-mCi group
	(*n* = 5)	(*n* = 5)
**Age (y)**	5.4 ± 2.3	4.8 ± 2.5
**Body weight (kg)**	5.1 ± 1.2	4.7 ± 1.3
**Sex (SF/CM)**	2/3	4/1
**Breed**		
Domestic shorthair	4	5
Persian	1	–
**T4 (μg/dL)**	2.83 (2.78–3.49)	2.89 (2.12–3.53)

Data for age, body weight, and sex are shown as mean ± standard deviation, while T4 is demonstrated as median and interquartile range. No significant differences were observed between the groups in terms of age, body weight, sex, and T4 levels (*p* > 0.05, Mann–Whitney U-test). SF, spayed female; CM, castrated male; T4, thyroxine.

### 3.2 TSR

**Figure 2:**
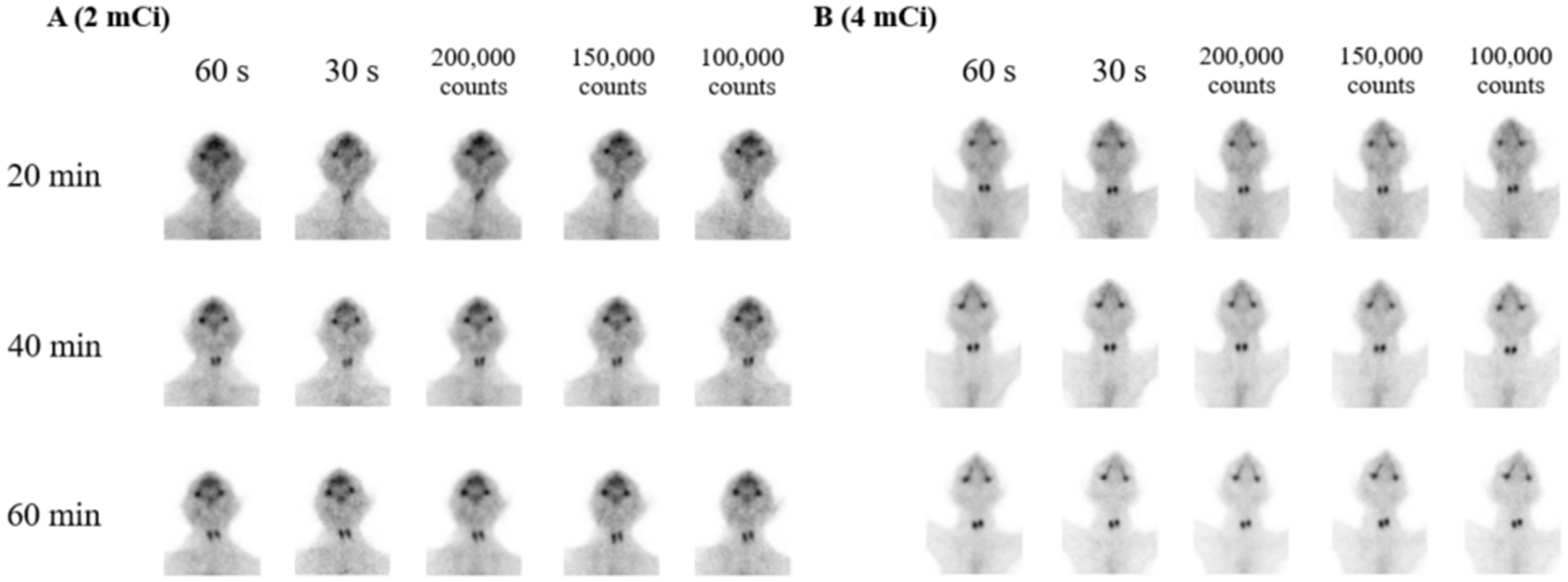
Serial scintigraphy images acquired at 20, 40, and 60 min after either 2 mCi **(A)** or 4 mCi **(B)** of technetium-99m pertechnetate injection using various acquisition conditions (100,000 counts, 150,000 counts, 200,000 counts, 30 s, and 60 s). The images are obtained from two selected cats: one from the 2 mCi group and one from the 4 mCi group.

In the comparison of TSR between the two dosage groups, no significant differences were identified for each acquisition condition at the three timelines (*p* > 0.05) except for TSR with 30 s of acquisition at 20 min post-injection (*p* = 0.043) (Figure 2 and Table 2). Additionally, the TSR was not significantly different according to the timeline and acquisition conditions (*p* > 0.05) (Supplementary Figure 1).

**Table 2:** TSR of 10 healthy cats evaluated according to timeline, acquisition condition, and injection dosage.

Timeline	Acquisition condition	2 mCi	4 mCi	*p*-value^a^
**20 min**	100,000 counts	0.884 (0.811–0.915)	0.889 (0.816–0.99)	0.436
	150,000 counts	0.961 (0.88–1.009)	0.937 (0.856–1.023)	0.796
	200,000 counts	0.905 (0.877–0.948)	0.972 (0.909–1.098)	0.353
	30 s	0.9 (0.857–1.021)	1.051 (0.959–1.094)	0.043^*^
	60 s	0.915 (0.899–0.925)	0.992 (0.879–1.041)	0.123
	***p*-value**^**b**^	0.904	0.080	
**40 min**	100,000 counts	0.797 (0.794–0.86)	0.731 (0.646–0.904)	0.971
	150,000 counts	0.871 (0.854–0.89)	0.679 (0.635–0.846)	0.529
	200,000 counts	0.865 (0.723–0.89)	0.643 (0.61–0.827)	0.436
	30 s	0.851 (0.775–0.897)	0.826 (0.709–0.93)	0.971
	60 s	0.825 (0.741–0.843)	0.732 (0.646–0.923)	0.912
	***p*-value**^**b**^	0.970	0.080	
**60 min**	100,000 counts	0.68 (0.624–0.793)	0.706 (0.552–0.742)	0.796
	150,000 counts	0.785 (0.673–0.825)	0.643 (0.533–0.762)	0.19
	200,000 counts	0.795 (0.76–0.799)	0.697 (0.579–0.711)	0.19
	30 s	0.707 (0.636–0.798)	0.68 (0.527–0.73)	0.436
	60 s	0.742 (0.729–0.788)	0.757 (0.592–0.799)	0.739
	***p*-value**^**b**^	0.581	0.850	

Data are expressed as medians (interquartile range). ^a^Comparison between the two groups; Mann–Whitney U test. ^b^Comparison among acquisition conditions: Kruskal–Wallis test. No significant differences were noted among the timelines (*p* > 0.05) using the Kruskal–Wallis test. Asterisks (^*^) indicate significant differences (*p* < 0.05).

### 3.3 TBR

Between the two dosage groups, significant differences in the TBR were noted only in three comparisons of 100,000 counts (*p* = 0.009) and 60 s (*p* = 0.029) of acquisitions at 40 min and 30 s (*p* = 0.003) at 60 min post-injection (Figure 2 and Table 3). Similar to the results for TSR, TBR did not differ significantly according to the timeline and acquisition conditions (*p* > 0.05) (Supplementary Figure 1).

**Table 3:** TBR of 10 healthy cats evaluated according to timeline, acquisition condition, and injection dosage.

Timeline	Acquisition method	2 mCi	4 mCi	*p*-value^a^
**20 min**	100,000 counts	2.132 (1.615–2.138)	2.079 (1.891–2.573)	0.315
	150,000 counts	2.119 (1.677–2.193)	2.033 (1.818–2.666)	0.481
	200,000 counts	2.062 (1.834–2.089)	2.324 (2.193–2.585)	0.075
	30 s	2.045 (1.818–2.114)	2.054 (1.946–2.146)	0.436
	60 s	2.009 (1.976–2.079)	2.045 (1.949–2.325)	0.247
	***p*-value**^**b**^	0.999	0.84	
**40 min**	100,000 counts	1.905 (1.763–2.391)	2.697 (2.567–2.837)	0.009^*^
	150,000 counts	1.871 (1.808–2.439)	2.466 (2.3–2.932)	0.063
	200,000 counts	2.131 (2.01–2.378)	2.334 (2.205–3.231)	0.123
	30 s	2.223 (2.215–2.487)	2.442 (2.309–2.815)	0.739
	60 s	1.98 (1.731–2.095)	2.272 (2.218–2.735)	0.029^*^
	***p*-value**^**b**^	0.475	0.514	
**60 min**	100,000 counts	1.99 (1.78–2.373)	2.414 (2.395–2.418)	0.075
	150,000 counts	2.708 (1.839–2.777)	2.461 (2.257–2.515)	0.481
	200,000 counts	2.413 (1.937–2.542)	2.476 (2.108–2.772)	0.393
	30 s	2.02 (1.813–2.167)	2.515 (2.39–2.675)	0.003^*^
	60 s	2.099 (2.037–2.596)	2.503 (2.388–2.598)	0.075
	***p*-value**^**b**^	0.626	0.995	

Data are expressed as medians (interquartile range). ^a^Comparison between the two groups; Mann–Whitney U test. ^b^Comparison among acquisition conditions: Kruskal–Wallis test. No significant differences were noted among the timelines (*p* > 0.05) using the Kruskal–Wallis test. Asterisks (^*^) indicate significant differences (*p* < 0.05).

### 3.4 Ambient radiation

**Figure 3:**
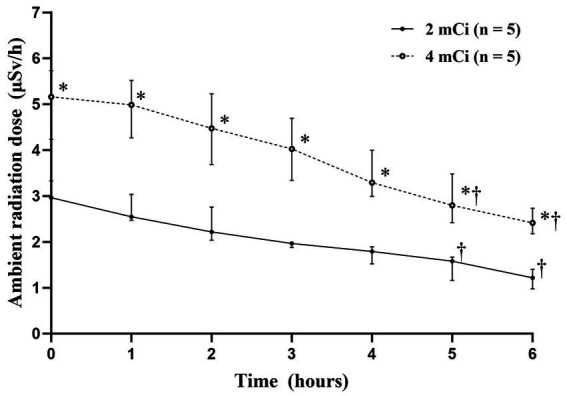
Median ambient radiation dose-time profiles (±interquartile range) after technetium-99m pertechnetate injection. * Statistically significant difference (*p* < 0.01) compared to the 2-mCi group using the Mann–Whitney U-test. † Statistically significant difference (*p* < 0.05) compared to 0-h using the Friedman test with Dunn’s multiple comparison.

After ^99m^TcO^−^_4_ injection, there was a significant difference in the ambient radiation doses between the two dose groups (*p* < 0.05). The radiation doses in the 4-mCi group were significantly higher than those in the 2-mCi group at each time point immediately after injection (*p* < 0.05) (Figure 3 and Supplementary Table 2). The ambient radiation dose decreased significantly over time in both groups (both *p* < 0.001). In particular, compared to 0-h, significant decreases were noted at 5-h in the 2-mCi (*p* = 0.009) and 4-mCi (*p* = 0.027) groups, and this decrease was maintained until 6-h in both groups (*p* < 0.05).

### 3.5 Surface radiation

**Figure 4:**
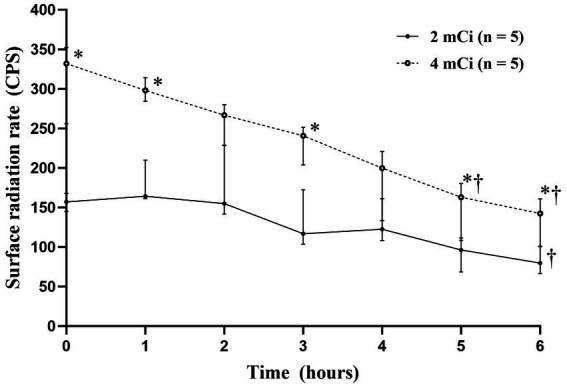
Median surface radiation dose-time profiles (±interquartile range) after technetium-99m pertechnetate injection. * Statistically significant difference (*p* < 0.05) compared to the 2-mCi group using the Mann–Whitney U-test. † Statistically significant difference (*p* < 0.05) compared to 0-h using the Friedman test with Dunn’s multiple comparison.

After ^99m^TcO^−^_4_ injection, there was a significant difference of the surface radiation doses between both groups (*p* < 0.05). The radiation doses in the 4-mCi group were significantly higher than those in the 2-mCi group at 0-h (*p* = 0.008), 1-h (*p* = 0.008), 3-h (*p* = 0.032), 5-h (*p* = 0.048), and 6-h (*p* = 0.032) (Figure 4 and Supplementary Table 3). The surface radiation dose decreased significantly over time in both groups (both *p* < 0.001). In particular, compared with 0-h, significant decreases were noted at 6-h in the 2-mCi group (*p* = 0.044) and at 5-h and 6-h in the 4-mCi group (*p* < 0.05).

### 3.6 Cumulative occupational radiation dose of veterinary staffs

**Figure 5:**
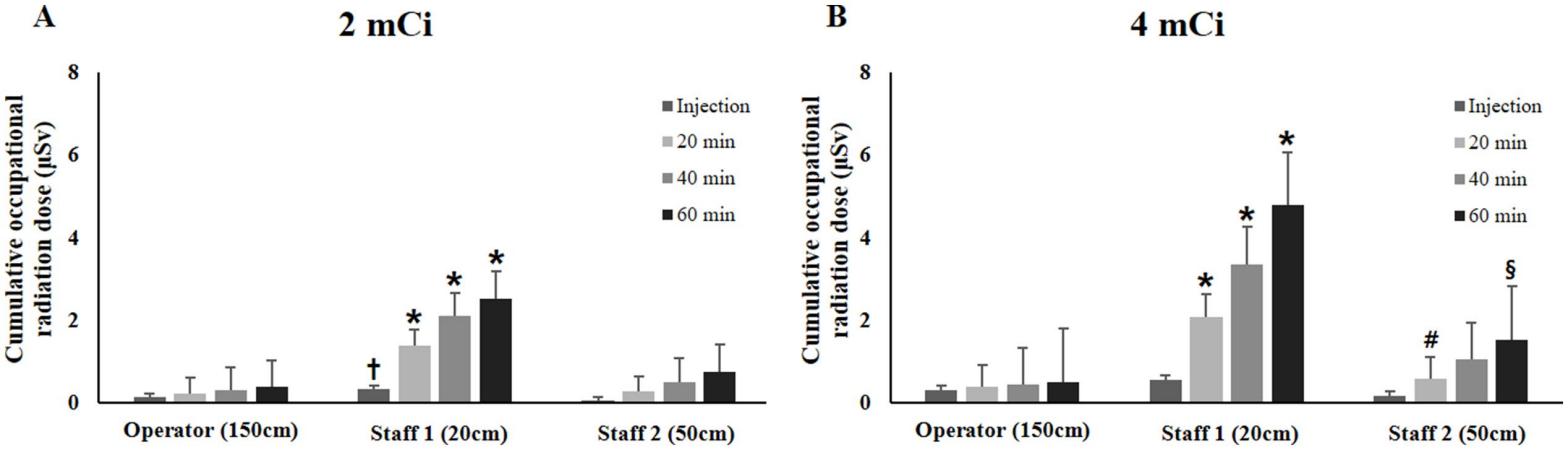
Comparison of mean cumulative occupational radiation dose (±standard deviation) of veterinary staffs (**A**, 2 mCi; **B**, 4 mCi). * Statistically significant difference (*p* < 0.05) compared to the operator using the Kruskal–Wallis test. † Statistically significant difference (*p* < 0.05) compared to staff 2 in the 2-mCi group using the Kruskal–Wallis test followed by Bonferroni correction. # Statistically significant difference (*p* < 0.05) compared to staff 2 in the 2-mCi group using the Mann–Whitney U-test. § Statistically significant difference (*p* < 0.05) compared to injection time of staff 2 in the 4-mCi group using the Friedman test with Dunn’s multiple comparison.

In the 2-mCi group, the cumulative radiation dose for staff 1 was significantly higher than that for staff 2 immediately after injection (*p* = 0.016). Furthermore, in both groups, the radiation doses for staff 1 were significantly higher than those for the operator from 20 to 60 min after the injection (*p* < 0.05) (Figure 5).

Changes in the cumulative radiation doses over injection time were not significant within each staff member for both dosage groups (*p* > 0.05). Compared with the value estimated after the injection, staff 2 had a significantly higher value at 60 min with the 4-mCi group (*p* = 0.019).

No significant difference in cumulative radiation dose was found between both dose groups (*p* > 0.05). However, in a paired comparison at 20 min, the radiation dose for staff 2 with the 4-mCi group was significantly higher than that with the 2-mCi group (*p* = 0.016).

## 4 Discussion

We evaluated the differences in the TSR and TBR across multiple variables, including administered activity, timeline, and acquisition conditions, using an SFOV camera. The results showed no notable differences in TSR and TBR between the two administered activities of ^99m^TcO^−^_4_, nor across acquisition conditions and timelines. Ambient and surface radiation doses were consistently higher in the 4-mCi group compared to the 2-mCi group, decreasing over time in both groups. Staff 1 consistently received higher cumulative radiation doses than did staff 2 and the operator.

### 4.1 Recommendations for thyroid scintigraphy in cats using SFOV gamma cameras

Based on these findings, when performing thyroid scintigraphy in cats using a SFOV gamma camera, it is recommended to minimize contact time with the radioactive cat by conducting the scan 20 min after the ^99m^TcO^−^_4_ injection with 100,000 counts. Additionally, to minimize radiation exposure by reducing the isotope amount, administering 2 mCi of ^99m^TcO^−^_4_ is recommended. These conditions can ensure adequate thyroid absorption to evaluate thyroid function while minimizing radiation exposure without the need for extra doses or time.

Because radiation exposure is determined by the dosage, distance, and time from the radiation source, it can be reduced by minimizing the radiopharmaceutical dose. However, insufficient doses may lead to inadequate thyroid absorption, resulting in inconclusive outcomes. Additionally, shortening acquisition conditions can minimize occupational exposure; however, excessively brief procedures may compromise image quality. Traditionally, thyroid scintigraphy with an LFOV gamma camera uses doses ranging from 2 to 6 mCi of ^99m^TcO^−^_4_ to achieve adequate TSR and TBR (3–7). In one study, 2 mCi of ^99m^TcO^−^_4_ were used to perform thyroid scintigraphy and achieved adequate TSR and TBR, although the image was acquired on 200,000 counts (23). In this study, quantitative differences in the radiopharmaceutical dose and acquisition conditions were evaluated during thyroid scintigraphy using an SFOV gamma camera. Most of the results aligned with the previously reported reference ranges (7, 24), and adequate image quality was notably achieved even with 2 mCi of ^99m^TcO^−^_4_ on 100,000 counts.

Despite the lower dose and shorter acquisition conditions, the absence of significant differences was attributed to the specific features of the SFOV gamma camera, such as minimizing the detector-to-target distance and restricting the field of view (8, 11). Furthermore, the mobility of the equipment makes it highly advantageous in cases in which patient movement is restricted. In conventional thyroid scans using a pinhole collimator, the thyroid image is magnified, posing challenges for accurate size assessment, and often necessitating the use of markers for compensation. However, with an SFOV gamma camera, images are acquired in the actual size, eliminating the need for markers, which is a notable advantage (11).

### 4.2 Radiation safety in thyroid scintigraphy for cats

To ensure the owner’s limit of a 1-mSv annual effective dose, the National Council on Radiation Protection and Measurements set the discharge criterion to <5 μSv/h (25). In the group receiving 2 mCi of ^99m^TcO^−^_4_, radiation levels remained below the discharge criterion from immediately post-administration onwards. However, the 4-mCi group met the discharge criterion starting from 3-h post-administration. After intravenous injection, ^99m^TcO^−^_4_ swiftly concentrates in various tissues like the salivary glands, choroid plexus, thyroid gland, gastric mucosa, and functioning breast tissue before being excreted through gastrointestinal and renal routes (26). To further minimize the exposure levels for the owner, cats should be encouraged to urinate before discharge (18), considering that the majority of radiopharmaceuticals used in nuclear medicine are eliminated through the urinary system (14).

Another radiation hazard is internal radiation exposure due to the ingestion, inhalation, or absorption of radioactive material. To predict the amount of radiation, studies on the surface radiation of radioiodine-treated cats were performed (27). In a previous study, surface radiation was measured using a gamma counter by wiping saliva-contaminated coats of cats treated for hyperthyroidism with 4 mCi of iodine-131, which resulted in radiation values ranging from 1.85 to 2.37 CPS (27). Conversely, in this study, immediately after injection, the surface radiation measured in the 4- and 2-mCi groups were 332 and 157 CPS, respectively. While a direct comparison is challenging owing to differences in equipment and radiopharmaceuticals, the radiation observed in this study was higher than that in a previous study (27). In contrast to the previous study wherein surface radiation was measured by wiping saliva-contaminated coats (27), in this study, surface radiation was obtained directly at a distance of 10 cm from the cat’s thyroid region. This resulted in radiation being measured not only from surface contamination but also from the cat’s body, which contained a higher concentration of radiopharmaceuticals. Therefore, considering the actual exposure situations in cats, the method used in this study could be a more accurate method for measuring surface radiation.

Cumulative occupational radiation doses in this study were measured using EPDs. Radiation exposure is commonly measured using thermoluminescent or optically stimulated luminescence dosimeters that provide cumulative doses to individuals over defined durations. However, EPDs can monitor radiation exposure in real time, enabling veterinary staff to adjust their conduct and procedures to comply with the as-low-as-reasonably-achievable principle. In this study, the differences in radiation exposure between the two dosage groups were mostly insignificant, but significant differences were found among the veterinary staff. Staff 1, who was the closest to the radiation source, experienced significantly higher radiation exposure than did the operator, who was the farthest from the source. Additionally, during the injection process, the operator was in closest proximity to the radiation source before the injection was completed, which could lead to higher exposure. Although the difference in radiation dose between the operator and Staff 2 did not reach statistical significance, the operator was more directly exposed during the injection process, which likely accounts for the observed higher level of irradiation compared to Staff 2, who had minimal involvement in the procedure. This result is consistent with previous research indicating increased exposure with closer proximity to the radiation source (16, 28).

The International Commission on Radiological Protection established guidelines specifying an annual occupational dose limit of 50 mSv, with an average of 20 mSv per year over 5 y (29). The annual dose limit for the public is set at 1 mSv (29). In this study, staff 1 received the highest cumulative dose 60 min after the injection of 4 mCi of ^99m^TcO^−^_4_, measuring 4.67 μSv, which is less than 0.01% of the annual occupational dose limit. Approximately 10,700 case per year must be examined to exceed the annual occupational dose limit. Considering the use of lead aprons during examinations and the increased frequency of scintigraphy under comparative experimental conditions, the anticipated radiation exposure of veterinary staff during thyroid scintigraphy using an SFOV gamma camera is expected to be extremely minimal (30). As veterinary scintigraphy has become more prevalent, there is an increased interest in cumulative occupational radiation doses for veterinary professionals.

In this study, the highest occupational radiation dose was 4.67 μSv, whereas in a previous study on the occupational radiation dose during feline renal scintigraphy, the maximum dose for a single case was assumed to be 25 μSv (18). This estimation was derived by measuring the ambient radiation levels at a distance of 50 cm and multiplying it by the operation time. However, this calculation did not fully consider the time spent in close proximity to cats, which occurs only during isotope administration and gamma camera imaging, neglecting the fact that most of the time, cats are separated from the staff. Consequently, there was a tendency toward overestimation.

This study has some limitations. First, the sample size of each group was small, which may have led to false-negative results (type II statistical error). Radiation exposure and thyroid scintigraphy data from more cats would optimize the safety and efficacy of the SFOV gamma cameras. Second, the efficacy of the SFOV gamma camera was evaluated without comparison with an LFOV gamma camera. According to studies in human breast cancer, SFOV BSGI allows for better separation of activity in the chest and abdomen, enhancing the detection of smaller and nonpalpable lesions compared to conventional LFOV gamma camera. Similarly, in cats, positioning them on the collimator during scans could enhance the detection of small thyroid lesions by separating them from thoracic and abdominal activity, thus increasing diagnostic accuracy (31). However, for an accurate comparison, it was essential to analyze the same subjects under identical conditions, but with different gamma cameras. Third, for dose-specific comparisons, it is necessary to conduct scintigraphy at various doses in the same subjects. However, this was not possible because the subjects in our study were client-owned, and additional visits would have led to reluctance from the owners. Last, a direct absolute quantitative comparison (such as counts per pixel) over varied doses was not performed because scintigraphy was conducted using multiple cats instead of a thyroid phantom, which absorbs a consistent amount of isotopes.

## 5 Conclusion

Satisfactory image quality for assessing the thyroid function in healthy cats can be achieved through thyroid scintigraphy using an SFOV gamma camera with relatively low doses and short acquisition conditions. The radiation exposure associated with this procedure poses minimal safety concerns. Therefore, the SFOV gamma camera may be a valuable tool for evaluating thyroid glands in cats.

## Data Availability

The original contributions presented in the study are included in the article/Supplementary material, further inquiries can be directed to the corresponding author.

## Supplementary material

SUPPLEMENTARY FIGURE 1 Serial scintigraphy images acquired at 20, 40, and 60 min after either 2 mCi or 4 mCi of technetium-99m pertechnetate injection using various acquisition conditions (100,000 counts, 150,000 counts, 200,000 counts, 30 s, and 60 s). The images represent the smallest cat from the 2 mCi group **(A)**, the smallest cat from the 4 mCi group **(B)**, largest cat from the 2 mCi group **(C)**, and the largest cat from the 4 mCi group **(D)**.
