# Supplementary figures and images for: Thyroid scintigraphy of healthy cats using small-field-of-view gamma cameras
Source: Front Vet Sci. 2024 Oct 21;11:1453441. doi: 10.3389/fvets.2024.1453441 (PMC11533270; doi:10.3389/fvets.2024.1453441)

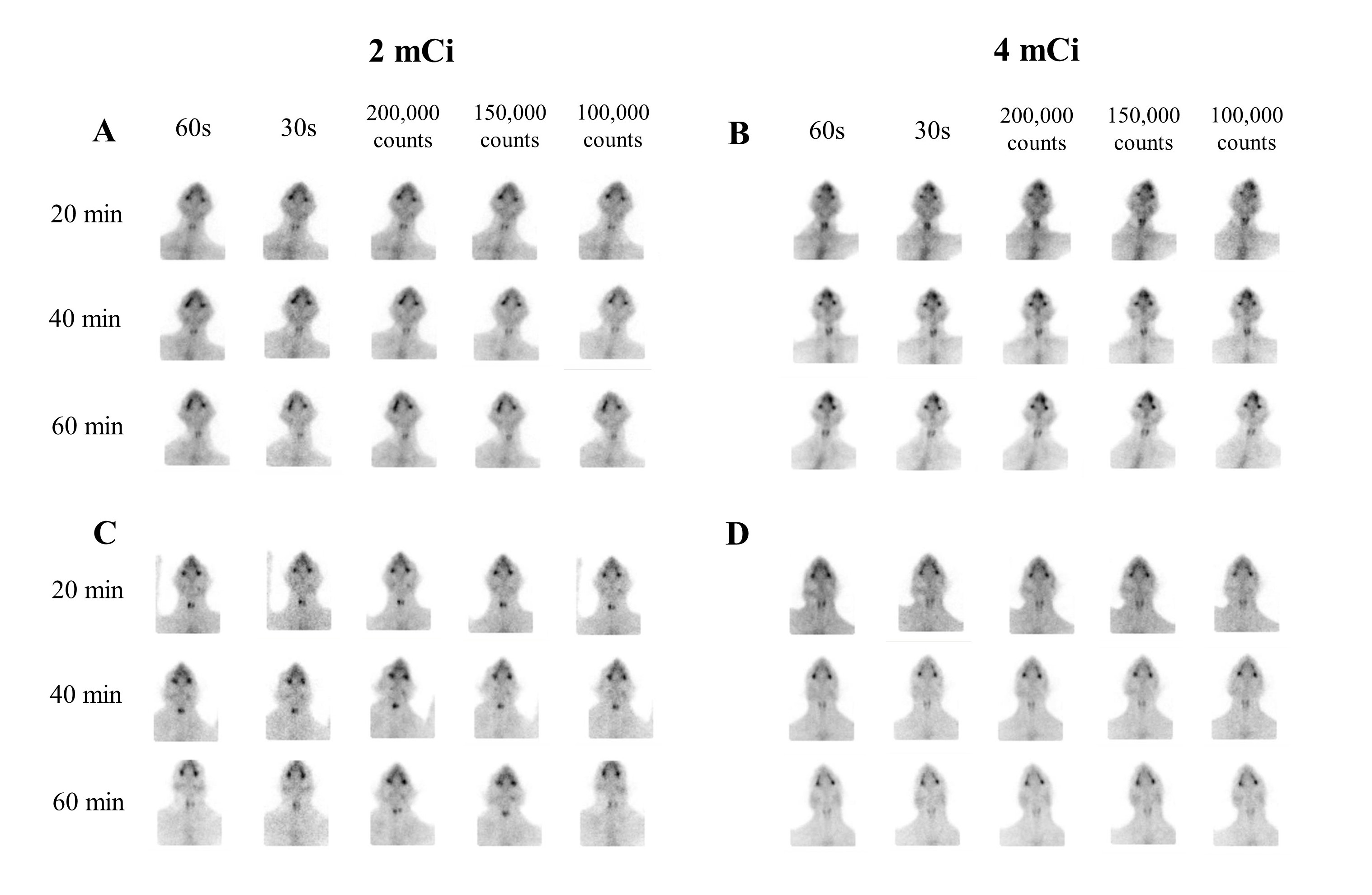

Supplement: SUPPLEMENTARY FIGURE 1 — Serial scintigraphy images acquired at 20, 40, and 60 min after either 2 mCi or 4 mCi of technetium-99m pertechnetate injection using various acquisition conditions (100,000 counts, 150,000 counts, 200,000 counts, 30 s, and 60 s). The images represent the smallest cat from the 2 mCi group (A), the smallest cat from the 4 mCi group (B), largest cat from the 2 mCi group (C), and the largest cat from the 4 mCi group (D). [file Image_1.jpeg]
