# Supplementary material for: Thyroid scintigraphy of healthy cats using small-field-of-view gamma cameras
Source: Front Vet Sci. 2024 Oct 21;11:1453441. doi: 10.3389/fvets.2024.1453441 (PMC11533270; doi:10.3389/fvets.2024.1453441)
Supplement: Supplementary file 2 [file Table_1.docx]

**Supplementary table 1**. Demographics of individual cat included in this study

|  |  | **Age**  **(y)** | **Body weight**  **(kg)** | **Sex**  **(SF/CM)** | **Breed** |
| --- | --- | --- | --- | --- | --- |
|  |  |  |  |  |  |
| **2 mCi** | **ID-1** | 3 | 4.08 | CM | DSH |
|  | **ID-2** | 8 | 6.50 | CM | DSH |
|  | **ID-3** | 6 | 4.37 | SF | Persian |
|  | **ID-4** | 7 | 4.35 | SF | DSH |
|  | **ID-5** | 3 | 6.29 | CM | DSH |
| **4 mCi** | **ID-6** | 3 | 6.87 | CM | DSH |
|  | **ID-7** | 4 | 3.63 | SF | DSH |
|  | **ID-8** | 3 | 5.01 | SF | DSH |
|  | **ID-9** | 5 | 4.15 | SF | DSH |
|  | **ID-10** | 9 | 3.85 | SF | DSH |

CM, castrated male; DSH, domestic short hair; SF, spayed female.
