# Supplementary material for: Thyroid scintigraphy of healthy cats using small-field-of-view gamma cameras
Source: Front Vet Sci. 2024 Oct 21;11:1453441. doi: 10.3389/fvets.2024.1453441 (PMC11533270; doi:10.3389/fvets.2024.1453441)
Supplement: Supplementary file 4 [file Table_3.docx]

**Supplementary table 3.** Results for surface radiation dose-time profiles after technetium-99m pertechnetate injection.

|  | **2 mCi** | **4 mCi** | ***p*-value** |
| --- | --- | --- | --- |
| **0h** | 157.0  (144.9-167.8) | 332.0  (255.9-352.3) | 0.008 |
| **1h** | 164.3  (161.2-209.7) | 298.0  (284.4-314.1) | 0.008 |
| **2h** | 155.0  (141.6-228.7) | 266.8  (228.3-279.9) | 0.127 |
| **3h** | 116.8  (103.5-172.4) | 240.7  (203.9-251.4) | 0.032 |
| **4h** | 122.6  (108.0-161.1) | 199.7  (133.4-220.9) | 0.198 |
| **5h** | 96.20^b^  (68.40-111.5) | 162.9^ab^  (108.4-180.4) | 0.048 |
| **6h** | 79.80^abc^  (66.30-100.6) | 142.5^ab^  (101.0-160.9) | 0.032 |
| ***p*-value** | <0.001 | <0.001 |  |

Data are expressed as medians (interquartile range). Comparisons within the same administered activity group over time were performed using the Friedman test with Dunn’s multiple comparison. Comparisons between the administered activity group (2 mCi and 4 mCi) at each time point were performed using the Mann-Whitney U test. a, *p* < 0.05 compared with 0h; b, *p* < 0.05 compared with 1h; c, *p* < 0.01 compared with 2h.
